# The Effort to Rationalize Antibiotic Use in Indonesian Hospitals: Practice and Its Implication

**DOI:** 10.1155/2023/7701712

**Published:** 2023-02-25

**Authors:** Selma Siahaan, Rukmini Rukmini, Betty Roosihermiatie, Pramita Andarwati, Rini S. Handayani, Ingan U. Tarigan, Tita Rosita, Rustika Rustika, Yuslely Usman, Lusi Kristiana

**Affiliations:** ^1^Organization Research for Health, The National Research and Innovation Agency-Indonesia, Jakarta, Indonesia; ^2^Centre for Health Financing and Decentralization Policy, Ministry of Health-Indonesia, Jakarta, Indonesia

## Abstract

An effective strategy for combatting AMR in Indonesia is to make the use of antibiotics in hospitals more rational with the help of an Antimicrobial Resistance Control Program (AMR-CP). This study aims to analyze the implementation of the AMR-CP in hospitals by conducting in-depth interviews with health professionals from ten hospitals and health officers of ten provincial health offices in ten different provinces and observation towards its documents. The sample location was selected by purposive sampling. Informants at the hospitals were hospital directors, chairmen of the AMR-CP team, chairmen of the medical committee, persons in charge of the microbiology laboratory, clinicians, nurses, clinical pharmacists, and those program managers at the provincial health offices who are responsible for administering antibiotics. Information is first collected and then a thematic analysis is applied along with triangulation to confirm the validity of information from multiple sources, including document observation results. The analysis is adapted to the framework of the system (i.e., input, process, and output). Results show that hospitals in Indonesia already have the resources to implement AMR-CP, including AMR-CP team and microbiology laboratories. Six hospitals examined also have clinicians trained in microbiology. Though hospital leadership and its commitment to implementing AMR-CP are favorable, there is room for improvement. AMR-CP teams organize routine activities for socialization and training, develop standard operating procedures (SOPs) for antibiotic use, antibiotic patterns surveillance, and bacterial mapping. Some obstacles to implementing AMR-CP policies are posed by the human resources, facilities, budget, antibiotics and reagent shortages, and clinician compliance with SOPs. The study concludes that there was an improvement in antibiotic sensitivity patterns, rational use of antibiotics, use of microbiological laboratories, and cost-efficiency. It recommends the government and healthcare providers continue to improve AMR-CP in hospitals and promote AMR-CP policy by making the regional health office of the hospital a representative of the regional government.

## 1. Background

The World Health Organization (WHO) declared that antimicrobial resistance (AMR) is one of the top ten threats to global health. The loss of effective antimicrobials increases both morbidity and mortality from infections. In 2019, there were an estimated 4.95 million AMR-related deaths. Lower respiratory tract infections account for more than 1.5 million resistance-related deaths. WHO's strategy for combatting AMR is leadership strengthening to address AMR, driving public health impact for the AMR response globally, research and development to improve access to value AMR prevention and care, and scrutinizing the AMR burden and AMR response in every country [1].

According to data from global antimicrobial resistance and use surveillance system gathered from 20 hospitals in Indonesia, there has been a growth in the percentage of some bacteria, such as *E. coli* and *K. pneumonia* that are resistant to antimicrobials. Carbapenems, fluoroquinolones, and third-generation cephalosporins are some of those [2]. In line with WHO's strategy, the Indonesian Ministry of Health issued a policy in 2015 on the sensible use of antibiotics. To use antibiotics “sensibly,” in their view, healthcare providers should consider where the disease originated and how resistant microbes were spread, i.e., improve the understanding and compliance of functional medical staff and other health workers in the wise use of antibiotics, increase the role of stakeholders in the field of infectious diseases treatment and the use of antibiotics, develop and improve the function of the clinical microbiology laboratory and other supporting laboratories related to infectious diseases treatment, improve clinical pharmacy services in monitoring the use of antibiotics, improve clinical pharmacology services in guiding the use of antibiotics, improve infectious cases treatment in an integrated multidisciplinary manner, carry out surveillance of patterns of antibiotic use and patterns of resistant microbes, and report them regularly.

Hospitals must be given careful consideration because they are a significant source of AMR. Researchers at Dr. Kariadi Hospital in Semarang and at Dr. Soetomo Hospital in Surabaya, both in Indonesia, reported that there was antimicrobial resistance *in S. aureus* and *E. coli*. Of the 3,995 *S. aureus* isolates, 25% were resistant to tetracycline. The level of *E. coli*'s resistance to gentamicin, cefotaxime, and ciprofloxacin was low in admission (4%, 2%, and 6%) but high in discharge (18%, 13%, and 22%), respectively [3]. This condition shows that the use of antibiotics is what regulates resistant microorganisms. Inpatients, surgical patients, and hospital emergency departments are at a high risk of a multidrug-resistant bacterial (MDRB) infection [4]. Reducing the consumption of broad-spectrum antimicrobials may alter the number of isolates with infection and antimicrobial resistance [5]. AMR was very dangerous for hospitalized patients. During hospitalization, 5–15% of all patients experienced healthcare-associated infections (HAIs). Some of these infections were sustained by multidrug-resistant (MDR) or even drug-resistant microorganisms, making therapeutic approaches very difficult [6, 7].

Several studies have shown that the incorrect application of antibiotic therapy was responsible for 30–50% of cases. In some cases, the right treatment was not indicated; in others, the choice of agent was wrong; and in others, the therapy lasted either too long or too short [8]. For these reasons, all hospitals should have an AMR control program staffed by health professionals from many different disciplines. Additionally, antimicrobial resistance control activities help hospitals to reduce the financial burden of antibiotics. This is particularly true for those who need treatment for infection and are covered by the National Health Insurance (NHS). The Republic of Indonesia's Ministry of Health issued Minister of Health (MOH) Decree no. 8/2015. This decree supported the Antimicrobial Resistance Control Program (AMR-CP) in hospitals and founded the National Committee for AMR control in 2015 to coordinate all AMR activities in the field of public health, even among professional organizations [9].

In this study, we discuss the extent to which AMR-CP in Indonesia helps to rationalize the use of antibiotics in hospitals. We conduct a comprehensive qualitative analysis, the results of which can be used as scientific evidence by the government, health care facilities, and medical practitioners. A more rational use of antibiotics can thereby be achieved, especially in Indonesian hospitals.

## 2. Method

This is a qualitative study that explores efforts to control antibiotic resistance in hospitals by rationalizing the use of antibiotics. Data were collected through in-depth interviews and observations. A written consent agreement was requested from the informants before the interviews began. The ethics committee of the National Institution of Health Research and Development thus issued ethical clearance for this study, which was conducted from February to October 2018.

### 2.1. Study Sites and Sample Locations

**Figure 1:**
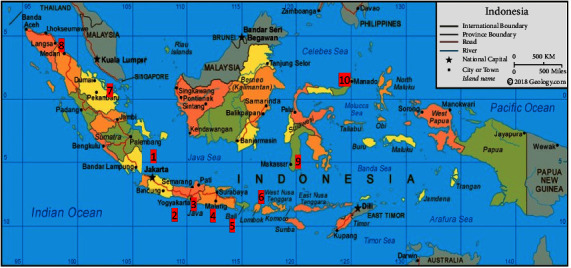
Study sites. Sources of picture: https://geology.com/world/indonesia-satellite-image.shtml.

The hospitals where data were collected were selected according to the following criteria: they have a microbiology laboratory, they have implemented an antimicrobial resistance control program, and clinical pharmacy services are already running. Based on these criteria, 10 hospitals were selected to serve as the sample of the survey. Almost all hospitals selected were public and teaching hospitals, and one hospital was both public and in a network of teaching hospitals.  Figure 1 shows the study sites of the hospitals.

### 2.2. Information Collected

The selection of informants refers to Minister of Health (MOH) Decree No. 8/2015 which regulates the composition of the AMR-CP team, especially those involved in the rational use of antibiotics both from the management and implementation sides, which are directly related to the use of antibiotics. Therefore, informants from the hospital include the chairman of the AMR control program team at the hospital, the chairman of the medical committee, the person in charge of the microbiology laboratory, and the specialist doctors who prescribe antibiotics frequently, such as specialist in internal medicine or pulmonary diseases, the nurse in charge of the ward, the clinical pharmacist, and the director of medical health services. The age of these informants ranged from 30 to 55 years old. They have at least one year of employment in the selected hospital and are related to the use of antibiotics. The interview duration was 1-2 hours per person and the audio recorded. The interview was conducted after research permit applications at the hospitals were granted. The research team and contact persons in each sampled hospital arranged the data collection process, including informants' availability and appropriate schedules. When the data collection began, the research team informed the hospital director of the research objectives and benefits. Effective coordination between the research team and the hospital management allowed the research data collection process to run smoothly. There were 62 informants who were interviewed. Some held multiple positions, for example, chairing the medical committee and serving as an expert in internal/pulmonary disorders, or simultaneously chairing the medical committee and the AMR-CP.

Before data collection, the researcher met with the head of the health office and obtained informed consent. Informants at the provincial health office included program managers in charge of the drug/antibiotics use program. There were 20 informants in the health offices who were interviewed.

The interview focuses on the policies about administering antibiotics to patients in hospitals, the implementation of AMR-CP, and the issues it raises. Several important inputs and outputs of AMR-CP implementation were observed, such as the decree for the establishment of the AMR-CP team, the availability of microbiology laboratories and clinical microbiology specialists, and the following documents: standard operational procedures (SOP), reports on the use of antibiotics, and program reports. All the interviewers were trained researchers who have been experienced in qualitative research interviews with a health-academic background, such as pharmacists and medical doctors. This study confirmed that there was no relation between the informants and the researchers. Most of the authors (6 out of 10) conducted interviews. Two researchers interviewed each informant: one acted as an interviewer and dialogued with the informant, and another documented both notes and recordings. All field notes were taken during the interview. The interviewing researcher transcribed the recording into a narrative. The interview process was only conducted once at a predetermined time because all questions had been answered completely, so the researchers did not repeat the visits and the transcript did not return to the informants. However, at the end of the study, researchers organized seminar that invited hospital informants and provincial health office representatives to provide preliminary study findings and ask for confirmation from the participants. If seminar participants submit confirmation, this confirmation will be discussed to reach an agreement and used as research results.

In this study, data were collected using observation lists and interview guides. Before collecting data and information, trials were conducted in two hospitals and one health office in two different areas from the study locations to test the instrument's validity and reliability. The trial results are processed, discussed within the team, and used to improve the instruments.

Interviews with informants were conducted privately at the hospital, in the workspace of each informant, using the Indonesian language without being accompanied by another person or translator. The interview might be interrupted when the informants suddenly need to attend to patients; the researchers would wait and resume the interview once the informants had concluded their interrupted duties. In this instance, the interview lasted longer. The informant was encouraged to provide accurate information based on the circumstances that existed in the hospital at the time of the interview. Given this significance, the researchers' guarantees of confidentiality and the requirement for information were explained before the interview.

### 2.3. Data Analysis

A theme-based analysis was conducted: first, the researchers transcribed the results of the interviews into the narrative; then they put them into categories and compressed them; finally, they entered the results into a matrix based on the theme and the hospital. The information in the matrix was color-coded to identify different themes, opinions, and languages. Important information was selected, and common issues were grouped together manually. The research team repeatedly discussed valuable information along with connectivity. Triangulation was carried out and checked with the results from the observations. We did all these analyses manually. The research team specifically looked for conformity between information from various sources. Once the objectivity of the data was confirmed, the results were discussed with experts and scholars in the fields of epidemiology and public health. On that basis, conclusions were drawn and recommendations for further study were made.

The information below is a summary report on 10 hospitals where informants were interviewed. The system concept of input, process, and output helped organize discussions of themes and the results of the observations. The themes for input are hospital policy/regulation, including its AMR-CP team and human resources; leader and management commitment; infrastructure;, and financing. The themes for the process are socialization, programs and activities, obstacles, and clinician compliance, while the output is AMR-CP's work results.

## 3. Result

All issues resulting from interviews and document observations refer to MOH Decree No. 8/2015 regarding AMR-CP in the hospital, with the results below.

### 3.1. Policy and Regulation

The results of in-depth interviews show that the hospital has carried out an antimicrobial resistance control program. The director of the hospital decreed that an AMR-CP team be formed in 2009 (at one hospital), in 2014 (at one hospital), in 2015 (at three hospitals), in 2016 (at two hospitals), and in 2017 (at two hospitals), and one hospital was not included in the decree. The AMR-CP team should consist of clinicians, nurses, clinical pharmacists, the person in charge of the microbiology laboratory, members of the infection prevention committee, and members of the pharmacy and therapy committee, with only one exception, and that was one hospital that located in DKI Jakarta Province did not comply to this policy. All AMR-CP teams thus report to the hospital director. However, all the informants were familiar with and understood the government regulation concerning the antimicrobial resistance control program in hospitals. The National Hospital Accreditation, furthermore, has required an AMR-CP team since 2017.

### 3.2. Leader and Management Commitment

Results from the in-depth interviews show that hospital leaders and management are committed to the implementation of AMR-CP, though some stated that their commitment still needs improvement. Information from management, practitioners, and the AMR-CP team is sometimes contradictory. Management stated that they had given full support to the implementation of AMR-CP, but the AMR-CP team stated it still needed improvement. The commitment was that management gave the chairmen and members of the AMR-CP team the opportunity to attend AMR training or workshops; supported teamwork needs, such as new computers, a secretariat, rooms, and staff; financed the programs that the team and its leaders attended; and recruited clinical microbiology specialists in the hospitals. On the contrary, the reason that leaders' and management's commitment still needs to be improved is that there are still AMR-CP teams that have no working space and lack funding for AMR-CP activities. The following is a statement submitted by the AMR-CP team chairman of one hospital:

“……… support for activity is good but insufficient financial support.”

### 3.3. Antimicrobial Resistance Control Program Resources and Reports

Table 1 shows the results from observations of the inputs and outputs of AMR-CP activities in hospitals: nine hospitals already have an AMR-CP team; all have microbiology laboratories; eight have SOPs for the use and restriction of antibiotics, reports on the use of antibiotics, and have made annual reports on AMR-CP activities; six have a specialist in microbiology; seven perform bacteria mapping.

### 3.4. Socialization of AMR-CP

The results showed that all hospitals carry out internal socialization activities, whether in the form of AMR-CP or general hospital meetings where one of the topics of discussion is AMR-CP. The socialization meetings were mostly aimed at clinicians, medical students who are doing internships, pharmacists, nurses, and other health workers. Meetings in some hospitals took the form of workshops and training. There is one hospital that conducts outreach to patients and their families to educate them about the rational use of antibiotics. The following is said by one chairman of AMR-CP in one hospital:

“……… We also conduct outreach to patients and their families about the rational use of antibiotics. We do this in a hospital meeting room and show them through exposure and questions and answers.”

The frequency of meetings varies, some hospitals hold them every 3 months, but others only 1-2 times a year. Meetings are more frequent if the hospital is seeking accreditation.

### 3.5. Implementation of the Program and the Hospital AMR-CP Team's Activity

The results show that the hospital has carried out AMR-CP activities, but its performance could be improved. The activities of some hospitals are still limited to AMR-CP preparation and socialization meetings. The activities at hospitals whose teams have been established for a longer time are more comprehensive and routine. Some respondents said that AMR-CP's activities were running slowly and even stuttering. The activities run more smoothly when the hospital is accredited. The following is the statement of the chairman of AMR-CP at one hospital:

“... for hospital accreditation, we were asked for the data of AMR surveillance or bacterial map, usually yearly data….”

Table 2 describes the AMR-CP team's inventory of activities in 10 hospitals that come from interviews with informants.

Table 2 shows activities that already have been done by all hospitals, i.e., organized regular AMR-CP team meetings, socialization activities, developing SOPs, and attending AMR-CP training. The least activities carried out by the hospital is evaluating the implementation of the AMR program.

The types of SOPs and the guidelines made for antibiotic control in hospitals are shown in Table 3.

Table 3 shows the types of SOPs and the guidelines made for antibiotic control in hospitals. All hospitals have made SOPs, but the types vary widely. Hospitals that have been implementing AMR-CP for a long time have made fairly complete SOPs, while hospitals that have just started AMR-CP only have one or two SOPs.

### 3.6. Obstacles to the Implementation of AMR-CP Activities

Obstacles reported in the interviews consisted of human resources, facilities, budget, availability of antibiotics, and the clinician's adherence to SOPs. Many hospitals stated that their AMR-CP budget was still insufficient. The budget for meetings, socialization, and stationery is relatively enough. There were hospitals that stated the budget for AMR-CP activities was sufficient but that funds for microbiological examinations were not. The AMR-CP team consists of doctors and other hospital health workers, who are busy; consequently, they do not coordinate well. Doctors still need refreshment training to understand antibiotic resistance better. Not all hospitals are equipped with facilities to support AMR-CP activities. The handling of specimens, the availability of reagents, and quality microbiological examinations still present problems. Shortages of antibiotics can cause antibiotics not to be used in accordance with the guidelines.

### 3.7. Clinician Compliance and Its Relationship to the Work Performance of the AMR Control Program

Based on the study result, the clinician's adherence to the guidelines/SOP and compliance with the results of antibiotic sensitivity tests are linked to the performance of the AMR-CP as described in Table 4.

Clinician adherence to the guidelines or SOPs for the use and restriction of antibiotics in hospitals varies widely: several of them were good, others were less compliant, and some hospitals had no evaluation. Compliance with the results of the sensitivity test is determined on the basis of the National Health Insurance treatment guidelines. These guidelines stipulate that, if a sensitivity test is not carried out, a higher line of antibiotics cannot be given. However, if it is still given to patients without proof of the sensitivity test results, NHS will not reimburse the treatment costs. This condition leads doctors to comply with the results of sensitivity tests but only for the use of higher-line antibiotics by means of second- or third-line antibiotics. The following stated by one medical health services director:

“…. clinician compliance here is also influenced by BPJS guidelines, because if they use high-line antibiotics without microbiological tests, they will not receive reimbursement............”

Although other factors may influence the work performance of AMR-CP, however, from Table 4, we can see there is a link between clinicians' compliance and work performance. The resulting evaluation of AMR-CP activities in hospitals is encouraging: the use of antibiotics was more rational than before; it led to a better quality of antibiotic use; there was a change in sensitivity patterns of antibiotics, increasing rational use of antibiotics; and the use of microbiological laboratories. In addition, there was greater cost efficiency for antibiotic treatment. However, three hospitals still need to evaluate the AMR-CP work performed.

### 3.8. Response of the Provincial Health Office on the AMR-CP Policy in Hospital

This study found that only the program managers of health offices in Java have a good understanding of MOH Decree No. 8/2015 concerning the antimicrobial resistance control program. It seems that other provincial health office managers know very little about the policy. Three out of ten health offices stated that they had introduced the AMR control program to hospitals in their area. The health offices in Java already had the data on hospitals in the area that our study was attempting to obtain, while others did not have any data, because they did not know about the AMR-CP activities in hospitals in their area.

The results of the interview likewise show that the health office is not yet committed to controlling AMR. This may be due to the fact that the health office itself does not fully understand AMR-CP. Furthermore, regarding the AMR policy, one officer in the health office stated:

“…… we have done socialization before, but rarely because it is not a priority program”

The health office thus confirmed the health officials had not been properly socialized about AMR-CP because the ministry of health quite often carries out AMR-CP socialization directly in the hospital without involving the health office. There was no funding for the AMR-CP program in the health office because it is not a priority program. In addition, MOH Decree No. 8/2015 did not provide the Provincial Health Office with technical guidelines for supervising hospitals. The health office has thus not monitored or evaluated the application of AMR-CP. The hospital AMR-CP team had also never submitted a report on AMR-CP activities to the health office. Several health offices said they could find out about the hospital's AMR-CP implementation if the hospital underwent accreditation, because they are usually invited to do so at that time. The statement below is from one of the program managers of the provincial health office:

“.... There are no specific activities, AMR-CP activities are included and are part of pharmaceutical activities for rational and national medicine for hospitals…”

## 4. Discussion

The framework system analysis starts with the input, including MOH Decree No. 8/2015 to form an AMR-CP team in the hospital. That policy aims to raise awareness and understanding, show commitment to solving the problem of antimicrobial resistance, and help create a national movement of hospitals, health professionals, communities, pharmaceutical companies, and local governments under the coordination of the Ministry of Health.

Thailand already started the Antimicrobial Resistance Containment and Prevention Program in 2011 [10]. Indonesia and Thailand are in the WHO Southeast Asia region; meanwhile, this region is dubbed a “global hub for AMR emergence” as it runs the highest risk for AMR emergence among all WHO regions in Asia [11]. It is expected the AMR policies in both countries may slow down the AMR.

The interviews confirm that all hospital informants understood the regulation of AMR-CP. Almost all hospitals have formed AMR-CP, which involves all relevant health professionals as mandated by the policy. The weakness of the policy is that it does not involve the local health office as a regional government representative in AMR-CP of the hospital, even though, based on the constitution, local health office is the regional health coordinator [12].

The hospitals in this study had the resources to implement AMR-CP policies, such as having a microbiology laboratory. Six hospitals had microbiology clinicians, and most hospitals had SOPs for using and restricting antibiotics. The AMR-CP team makes reports and documents about the use of antibiotics, including maps of bacteria. These reports, which consist of pathogenic bacteria and antibiotic resistance, can be used to make local policy decisions by management together with the AMR-CP team. These decisions must be made according to their respective conditions, especially the development of infection prevention and control programs [13], access to essential antibiotics, and research on and development of new vaccines and antibiotics [14, 15].

The analysis of commitment that informants believed the leadership and management had to implement AMR-CP was encouraging, though there is room for improvement. A high degree of commitment is shown in facilitating the AMR-CP team to participate in training, appropriate human resources, support infrastructure, and finance AMR-CP activities. Informants who stated that their commitment needed to be improved felt that there was a lack of AMR-CP workrooms and funding for AMR-CP activities. The hospital director, together with the AMR-CP team, should therefore set measurable work goals for the AMR-CP team to accomplish and facilitate its successful completion of them. Targets need to be made so that AMR-CP programs and activities can be clearer and more focused on achieving goals [16].

All hospitals promoted the socialization of AMR-CP policy, especially among health workers and internship doctors and among medical college students in the form of workshops and training. Afari-Asiedu et al. [17] reported that, of the 13 studies they systematically reviewed, 69.2% claimed to implement measures designed to make the AMR program more effective. These forms of intervention include educational and refreshing meetings, local consensus, and involving stakeholders [15]. The results of our study also reported that one hospital carried out socialization for patients and their families as well [17, 18]. The success of AMR-CP depends not only on the knowledge and expertise of health care providers but also on patients and their families. Research has shown that the knowledge that the patient's family has about the use of antibiotics is sufficient, while knowledge about antibiotic resistance is low [19]. Other studies found that respondents with low levels of knowledge about antibiotics had a higher likelihood of self-medication, and conversely, those who have good knowledge about antibiotics have a good awareness of the problem of AMR [20, 21]. It is therefore important that hospitals provide comprehensive patient education.

Almost all of the informants said that AMR-CP activities in hospitals had not been optimal. Table 2 shows some of the activities that the AMR-CP team should carry out in only a few hospitals. It is also noteworthy that only six hospitals made AMR-CP activity reports and submitted them to the hospital leaders. Many institutions in Indonesia are weak on evaluation and reporting, especially in the public sector. Hospital leaders and those external to the hospital, therefore, should begin to challenge them. Hospital leaders should create a reliable system for monitoring AMR-CP activities in hospitals so that the team feels more committed to it [15, 22]. Hospital accreditation, on the contrary, can provide pressure from outside the hospital. Hospitals that have just undergone accreditation will show better performance, as the antimicrobial stewardship standard is part of hospital accreditation requirements [23–25].

Seven hospitals performed bacterial mapping, and the other three hospitals never did, because AMR-CP had only been established very recently and their microbiology laboratory was relatively new (±one year). Bacterial maps are important, as they make data on bacterial epidemiology and patterns of antibiotic resistance in Indonesia available for healthcare professionals to evaluate. Bacterial mapping thus helps to improve the AMR-CP program and provides a reference for the development of empirical treatment guidelines, especially in hospitals that still have limited local data. AMR surveillance studies in 31 hospitals showed slightly different patterns of antibiotic susceptibility, depending on what type of hospital and which clinical specimens [26].

As far as the types of SOPs and guidelines used to control antibiotics are concerned, the study shows that all hospitals made SOPs, but the types vary greatly. The results of another study stated that there were antibiotic guidelines in five out of six general hospitals in Jakarta surveyed, and that adherence to the guidelines was only around 52.2%. The prescribing and discontinuation of antibiotics and how long they are used for were unplanned and not well documented. Furthermore, documentation of the use of antimicrobials is very poor, and adherence to the guidelines is very low [27]. Based on these circumstances, we recommend that the Ministry of Health cooperate with the Council of Medical Specialty Societies to develop guidelines and SOPs that are general in nature and must be owned by hospitals as a minimum, which are required for AMR-CP which can be used as a reference for every hospital, for example, guidelines for controlling AMR, SOP for selecting empiric and definitive antibiotics, SOP for preparing and discontinuing antibiotics, and SOP for approval for antibiotics, whereas SOP guidelines for using antibiotics at the department/installation/unit level would be best when locally tailored based on the pattern of germs and the availability of data on resistance patterns.

Obstacles to implementing AMR-CP policies were presented by limitations in human resources, facilities, or budget, as well as the availability of drugs, antibiotics, reagents for sensitivity tests, and clinician compliance with SOPs. Not all hospitals are equipped with facilities to support AMR-CP activities. Hospitals on Java have better facilities and human resources as this island is the most developed area in Indonesia [28]. The handling of specimens and the quality of microbiological examinations were also still a problem. Shortages in drugs and antibiotics can cause antibiotics to circulate that are not in accordance with the guidelines. Likewise, research has recently shown that the implementation of AMR-CP activities has not been optimal because of various program challenges. Some of these challenges include lack of funding, commitment, and coordination with internal hospitals that have not been well established, inadequate infrastructure, and problems with referral patients that have already experienced resistance [2]. Another study found that AMR control was implemented better in government hospitals than in private hospitals. Problems in private hospitals are ineffective communication, limited resources, lack of antibiotic guidelines, lack of coordination between organizations, lack of supervision from the government, and lack of motivation to implement policies [29].

Strict compliance with guidelines must be observed, in addition to the rational prescribing of antimicrobials [30]. Results show that clinician adherence to the guidelines or SOPs that regulate the use and restriction of antibiotics in hospitals varies widely. The compliance of several hospitals was favorable, others were obedient, some were less compliant, and some hospitals have not even been evaluated. Clinician compliance with sensitivity test results is improving because of NHS regulations. These regulations require that a sensitivity test be administered before high-line antibiotics are prescribed. If antibiotics are still given to patients without evidence of sensitivity test results, NHS will not reimburse them. It is thus very important that clinicians apply the sensitivity test and adhere to the SOP when prescribing antibiotics, as these practices help to prevent multidrug resistance. In 2014, Indonesia launched NHS to help make universal health coverage possible.

Research in Chinese hospitals has shown that infection with multidrug-resistant bacteria (MDRB) has become a severe clinical and therapeutic dilemma. The government put pressure on hospitals to implement AMR, establish proper infrastructure for the rational use of antibiotics, and develop a professional team to ensure AMR control [31]. Meanwhile, the results of this study indicate that only six hospitals monitored the emergence and spread of multiresistant microbes in hospitals, and only one hospital developed guidelines for multidrug resistance organisms. This should be a concern for the government and hospitals.

The study results showed that AMR-CP activities in hospitals lead to encouraging results, such as changes in antibiotic sensitivity patterns, increased rational use of antibiotics, increased use of microbiological laboratories, and greater cost-efficiency. However, three hospitals still have not evaluated the AMR-CP team's ability to control antibiotic resistance in hospitals. Implementing an antibiotic stewardship program, leadership and management commitment, as well as the expertise of the team members, and an effective antimicrobial use policy helped to increase the rational use of antibiotics, decrease the prevalence of resistance, and provide significant economic benefits [32–34]. Therefore, AMR-CP should be supported and expanded so that antibiotics are used equally rationally throughout the hospital [35]. Planning of AMR-CP activities and budgeting is needed to raise commitment and overcome a shortage of resources and funding.

Something else this study found is that most provincial health offices do not fully understand AMR-CP in hospitals or what it does. Health offices on the island of Java, by contrast, have a better understanding and better data on hospitals in their area that have conducted AMR-CP (three provinces). Other health offices, however, do not have data and do not know about what AMR-CP does in the hospitals in their area. Coordination between the Health Office and hospitals is therefore the key to raising awareness, improving understanding, and expressing a shared commitment to AMR so that it becomes a national movement as the government mandated in Minister of Health Decree No. 8/2015.

### 4.1. Limitation of the Study

The availability of the hospital samples that fulfilled the criteria was limited. Accordingly, more hospitals are located in Java than in other areas, and all hospitals are public and network of teaching hospital, which may produce different results if they are not teaching hospitals.

## 5. Conclusion

The work performed by Hospital Antimicrobials Resistance Control Programs started to improve the rational use of antibiotics and drug cost efficiency in hospitals; however, it is still too early to say that AMR control in hospitals has had an impact on reducing AMR. Therefore, the government and healthcare providers should continue to strengthen AMR-CP in hospitals. The hospital management should also make available antibiotics and reagents for sensitivity tests in accordance with the hospital's needs through good drug planning and procurement. Hospital leaders and the AMR-CP team should set measurable objectives and monitor and evaluate AMR-CP activities, so that AMR-CP can more readily achieve its objectives. AMR-CP policy must be improved by making the regional health office of the hospital a representative of the regional government and thus also by turning AMR-CP into a national movement.

## Figures and Tables

**Table 1:** Observation in 10 hospitals: resources and performance AMR-CP team 2018.

No.	Resource and report components	AAA hosp	BBB hosp	CCC hosp	DDD hosp	EEE hosp	FFF hosp	GGG hosp	HHH hosp	III hosp	JJJ hosp
1	Have a AMR-CP team decree issued by the director of the hospital	√	√	√	√	√	—	√	√	√	√
2	Availability of microbiology laboratory	√	√	√	√	√	√	√	√	√	√
3	Presence of a clinical microbiologist	—	—	√	√	√	—	√	√	—	√
4	SOPs for the use and restriction of antibiotics	—	√	√	√	√	—	√	√	√	√
5	Reports on the use of antibiotics	—	√	√	√	—	√	√	√	√	√
6	Annual reports on AMR-CP activities	—	√	√	√	—	—	√	√	√	—
7	Bacterial map	—	√	√	√	√	—	√	√	√	—

**Table 2:** AMR-CP team activities in 10 hospitals in Indonesia, in 2018.

No.	AMR-CP team activities	Number of hospitals implementing
1	Regular AMR-CP team meetings	10 hospitals
2	Socialization	10 hospitals
3	Developing SOPs on AMR control in the hospital	10 hospitals
4	SOP evaluation and revision	4 hospitals
5	Integrated infectious disease management	4 hospitals
6	Surveillance of antibiotic use patterns	7 hospitals
7	Education and training	10 hospitals
8	Research on AB resistance control	5 hospitals
9	Reporting activities to directors	7 hospitals
10	Evaluation of AMR program implementation	3 hospitals
11	Evaluation of the use of antibiotics	
	(i) Geysen method	7 hospitals
	(ii) DDD method	7 hospitals
12	Monitoring the spread of multiresistant antimicrobial	6 hospitals

**Table 3:** Types of guidelines and standard operating procedures (SOPs) that have been prepared by the AMR-CP team in 10 hospitals in Indonesia, in 2018.

AMR-CP team activities	Number of hospitals implementing
(i) Guidelines for use and restriction of antibiotics	8 hospitals
(ii) Selection of antibiotics empirical and definitive	6 hospitals
(iii) Guidelines for AMR control	5 hospitals
(iv) SOP for antibiotic preparation	4 hospitals
(v) SOP for stopping antibiotics	2 hospitals
(vi) SOP for approval of antibiotics that not listed in the national formulary	2 hospitals
(vii) SOP for prophylactic antibiotics, MRSA case guidelines, SOP for controlling MDRO, and antibiotic pocketbook	1 hospital

**Table 4:** Clinicians' compliance with guidelines for the use and restriction of antibiotics and antibiotic sensitivity tests and link to AMR-CP team performance in 10 hospitals, 2018.

No.	Hospital name	Clinicians' compliance		AMR-CP team performance
		Guidelines for use of antibiotics and restriction of antibiotics	Antibiotic sensitivity test results	
1	AAA hospital	Compliance was quite good because it is part of the hospital accreditation assessment	Clinicians sometimes did not use the antibiotics test results, and they often did not ask for a sensitivity test	In general, there is a decrease in the use of antibiotics, although the hospital has not explicitly evaluated the performance of the AMR-CP. Good hospital compliance due to accreditation requirement, but sustainability needs to be improved. This can be seen from the fact that clinicians rarely ask for antibiotics sensitivity tests. However, the impact of SOP on antimicrobial resistance can still be seen in reducing the use of antibiotics
2	BBB hospital	Clinicians' adherence to antibiotic use guidelines was good	Clinicians' compliance with sensitivity test results because it is in line with NHS treatment guidelines	Improvements in the result of sensitivity pattern based on Geysen qualitative analyses
3	CCC hospital	Clinician compliance is about 50% with the antimicrobials' guidelines and SOPs, but clinicians are quite obedient to clinical practice guidelines and pathways	Clinicians have started to comply with the use of antibiotics by considering the culture results	Work performance has never been measured, but the use of antibiotics is much more well-arranged now. There is progress in terms of the use of antibiotics based on the results of sensitivity tests and reducing the use of antibiotics for prophylaxis
4	DDD hospital	Compliance is getting better, although some still have not obeyed. Those who have not complied more adhere to international guidelines	Compliance with sensitivity test results was perfect	There was significant progress in microbiologist laboratory services due to the hospital implementing AMR-CP. Analyses with the Geysen method showed that the rational use of antibiotics had reached 80%. Thus, it leads to the cost efficiency of antibiotics to billion rupiahs (hundred thousands dollars). At the same time, antimicrobial treatment quality is improving as a reference
5	EEE hospital	Clinical compliance is still lacking	Compliance with sensitivity test results has not been evaluated	The work performance of AMR-CP has never been evaluated, but since the implementation of AMR-CP in the hospital, the use of antibiotics is more controlled and rational
6	FFF hospital	It has not been evaluated	It has not been evaluated	The work performance of AMR-CP has not been seen, as the program just started around one year
7	GGG hospital	Not all clinicians have implemented it because uniform understanding is not easy. They were generally accepted, especially among young clinicians	Compliance with sensitivity test results has not been evaluated	Even though there were no comprehensive evaluations on AMR-CP, the rational use of antibiotics increased; conversely, the irrational use of antibiotics decreased by 60%. In addition, there is an improvement in the quality of the use of antibiotics
8	HHH hospital	Compliance with the guidelines was good	Compliance with sensitivity test results was good, mainly because it is in line with NHS guidelines. If it is outside NHS, then the claim will not be paid	There was a decrease in the use of antibiotics without a diagnosis of infection, from 80% to 77.7%, and the effectiveness was better, ranging from 70 to 80%. The cost aspect leads to being more efficient because the cost is lower. Overall, there were better quality and cost control
9	III hospital	Compliance with the guidelines has been established but still needs to be improved	Some clinicians still need to comply with the results of sensitivity tests, and there are still doctors who use antibiotics in the long term without supporting data such as sensitivity test results	There was a change for the better quality of antibiotic use. Likewise, there was an improvement in antibiotic sensitivity and a decrease in multiresistant antimicrobials. There was also a decrease in the incidence of MRSA
10	JJJ hospital	There is no clinical compliance assessment yet	It has not been evaluated	Although not yet evaluated, AMR-CP has a cost-efficiency impact

## Data Availability

The data used to support the findings of this study may be released upon application to the National Institute of Health Research and Development Republic of Indonesia transformed to be the Agency for Health Policies Development, Ministry of Health, Indonesia, which can be contacted via tu.kabadan@gmail.com.
